# Comparative Study of Glycemic Control and Treatment Satisfaction in Patients With Type 1 Diabetes on Sensor-Augmented Insulin Pump, Insulin Pump, and Multiple Daily Insulin Injections: A Cross-Sectional Study

**DOI:** 10.7759/cureus.94771

**Published:** 2025-10-17

**Authors:** Ali A Alshahrani, Fajr Mutairi

**Affiliations:** 1 Endocrinology and Metabolism, King Abdulaziz Medical City, Riyadh, SAU

**Keywords:** diabetes type 1, glycemic control, insulin multiple daily injections, insulin pump, patient satisfaction

## Abstract

Background: Patients with type 1 diabetes mellitus (T1DM) depend on lifelong insulin therapy. In recent decades, advances in diabetes technology have introduced insulin pumps and continuous glucose monitoring (CGM) systems, offering new approaches to disease management. Despite these innovations, evidence regarding their impact on glycemic control and patient satisfaction within the Saudi population remains scarce. This gap is important, as cultural, lifestyle, and genetic factors may influence treatment outcomes.

Patients and methods: A cross-sectional study was conducted at the Endocrine Center, King Abdulaziz Medical City, Riyadh, Saudi Arabia. Eligible participants were patients aged 14 years and older with T1DM who had been on one of the following regimens for at least six months: a sensor-augmented insulin pump (MiniMed 640G or 740G, Medtronic, Dublin, Ireland), an insulin pump without a sensor (Paradigm VEO 754, Medtronic), or multiple daily insulin injections (MDIs).

Results: The study enrolled 196 patients, the majority of whom were female (76.5%), with ages ranging from 14 to 55 years. Over half of the cohort (52.5%) were treated with MDI, while 25% and 8.2% used sensor-augmented pumps and non-sensor pumps, respectively. Regression analyses demonstrated that patients using sensor-augmented pumps achieved significantly lower glycosylated hemoglobin (HbA1c) levels compared to those treated with MDI. In addition, sensor-augmented pump users reported markedly higher satisfaction scores. The frequency of educator visits explained approximately 20% of the variability in satisfaction (R² = 0.202).

Conclusions: Sensor-augmented insulin pumps provide superior outcomes compared with MDIs in patients with T1DM, offering both improved glycemic control and greater patient satisfaction. These findings underscore the importance of integrating advanced technologies into diabetes care, particularly in populations with unique cultural and lifestyle characteristics.

## Introduction

Type 1 diabetes mellitus (T1DM) is a chronic condition characterized by insulin deficiency resulting from the destruction or absence of pancreatic β-cells, leading to persistent hyperglycemia [1-4]. This autoimmune disease is particularly prevalent among children and adolescents worldwide and is often associated with both acute and long-term complications [5].

A large multi-cohort study conducted in Finland demonstrated significant improvements in life expectancy among individuals with T1DM over recent decades. However, despite these advancements, life expectancy for this population remains approximately 9.9 years shorter than that of the general population [6]. Management of T1DM universally requires lifelong insulin therapy, typically administered through subcutaneous injections [7].

In the past decade, innovative technologies such as insulin pumps, sensor-augmented insulin pumps (SAIPs), and hybrid closed-loop systems have been introduced, offering more precise glycemic control [4,6]. These technologies represent a significant shift in the management of T1DM, moving beyond traditional multiple daily insulin injections (MDIs).

Tight glycemic control is critical for reducing the risk of both microvascular and macrovascular complications in patients with T1DM [4]. Nevertheless, despite advances in treatment, many patients continue to experience such complications. Clinical trials have demonstrated that SAIP therapy leads to a rapid and sustained improvement in glycated hemoglobin (HbA1c) levels [8].

In one such trial, patients previously on MDI therapy were randomized to receive either continued optimized MDI or transition to SAIP therapy. After one year, HbA1c levels decreased to 8.1% in the MDI group and to 7.5% in the SAIP group, with a statistically significant difference (p < 0.001) [9]. During an optional six-month extension, patients in the SAIP group continued their therapy, while those initially on MDI were allowed to switch to SAIP. Results showed that HbA1c improvements in the original SAIP group were sustained for 18 months. Additionally, patients who switched from MDI to SAIP also demonstrated significant reductions in HbA1c compared to their 12-month baseline values [10]. The main aim of this study is to compare glycemic control and treatment satisfaction among patients with type 1 diabetes using SAIPs, insulin pumps without sensors, and MDI. The specific objectives are to assess differences in HbA1c levels among the three treatment modalities, evaluate treatment satisfaction scores, and explore associations between glycemic control, satisfaction, and clinical variables such as educator visits and carbohydrate-counting knowledge.

## Materials and methods

The study was conducted at the Endocrine and Diabetes Center of King Abdulaziz Medical City in Riyadh, a major tertiary referral center for T1DM. The type of insulin modality used by patients was determined based on patient readiness and other clinical eligibility factors assessed at the center. To be included in the study, participants had to be adolescents or adults aged 14 years or older. They were required to have been using one of the following for at least six months: a SAIP (MiniMed 640G or 740G (Medtronic, Dublin, Ireland) with suspend on low and pre-low features, paired with the Guardian Connect Sensor 7 or Libre 2), an insulin pump without a sensor (Paradigm VEO 754, Medtronic), or MDI.

Exclusion criteria comprised patients with cognitive impairments; those using oral hypoglycemic agents other than metformin (prescribed for clinical indications such as polycystic ovary syndrome); individuals with psychiatric diagnoses or on psychiatric medications; and patients younger than 14 years. Additionally, patients on MDI who used sensor-based continuous glucose monitoring (CGM) devices with real-time glucose alarms, as well as those using hybrid closed-loop pumps, were excluded from the study.

The study sample reflects patients typically managed at tertiary diabetes care centers, thereby enhancing the generalizability of the findings to similar specialized clinical settings. Adult patients and guardians were invited to complete a self-administered questionnaire electronically via Google Forms (Google, Mountain View, CA). This questionnaire was designed to collect demographic and habitual data, including age, sex, educational level, occupation, marital status, place of residence, smoking status, and body mass index. In addition, diabetes-related information was obtained, including disease duration, co-morbid conditions, such as hypertension, cardiac diseases, ischemic heart disease, dyslipidemia, thyroid disorders, and other endocrine conditions. The presence of diabetic complications, such as retinopathy, nephropathy, neuropathy, and diabetic foot, was also recorded.

From the Google Sheets database, we extracted each patient’s most recent HbA1c value within the preceding three months, the number of visits to a diabetes educator since diagnosis, and the frequency of diabetic ketoacidosis (DKA) admissions over the past year.

To assess treatment satisfaction, the Arabic version of the Diabetes Treatment Satisfaction Questionnaire (DTSQs) was used. This tool was adapted from the original English version developed by Prof. Clare Bradley [11]. The DTSQs is a validated instrument designed to measure satisfaction with diabetes treatment across different therapeutic modalities. It consists of eight items: six assess general satisfaction with the current treatment (each scored from 0 = very dissatisfied to 6 = very satisfied), and two evaluate the perceived frequency of hyperglycemic and hypoglycemic episodes (scored from 0 = never to 6 = most of the time). The total treatment satisfaction score is calculated by summing the six satisfaction items, with higher scores indicating greater satisfaction [11].

The English version of the questionnaire is included in the Appendix. Use of the Arabic version was authorized under license (CB1015) granted to Dr. Ali A. Alshahrani by the Health Psychology Research Unit, Royal Holloway, University of London.

Ethical approval for the study was obtained from the Institutional Review Board of King Abdullah International Medical Research Center (Reference No. RC20/120/R), in accordance with the principles outlined in the Declaration of Helsinki. Additionally, written permission was obtained from the Director of the Endocrine and Diabetes Center at King Abdulaziz Medical City. All adult participants and guardians provided written informed consent prior to participation. Confidentiality was strictly maintained, and all data were used exclusively for research purposes.

Quantitative continuous variables are presented as means ± standard deviations, while categorical variables are summarized using frequencies and percentages. Group differences were assessed using chi-square (χ²) tests for categorical variables and one-way analysis of variance (ANOVA) for comparisons involving more than two groups. When the ANOVA test yielded significant results, Tukey’s post hoc test was performed for pairwise comparisons between groups. A p-value of less than 0.05 was considered statistically significant. To control for potential confounders, multiple linear regression analysis was conducted. Data entry and statistical analyses were performed using IBM SPSS software version 28 (IBM Corp., Armonk, NY).

## Results

The study included 196 patients with type 1 diabetes, the majority of whom were female (150 patients, 76.5%). Participants’ ages ranged from 14 to 55 years, with a mean age of 23.7 ± 9.2 years. Most patients resided in urban areas (169, 86.2%), were unmarried (162, 82.7%), and were not currently employed (153, 78.1%). A substantial portion, 83 patients (42.4%), had completed university or postgraduate education. Only a small number reported a history of smoking, either current or past, accounting for 10 individuals (5.2%). Regarding body weight, over one-third of the participants were classified as overweight (42, 21.4%) or obese (30, 15.3%) (Table 1).

**Table 1:** Personal characteristics of the participants (n = 196). Descriptive statistics are presented as frequencies and percentages.

	Total (N = 196), N (%)	Insulin pump with sensor (N = 77), N (%)	Insulin pump without sensor (N = 16), N (%)	Multiple dose insulin inject (N = 103), N (%)
Gender				
Male	46 (23.5)	15 (19.5)	5 (31.3)	26 (25.2)
Female	150 (76.5)	62 (80.5)	11 (68.8)	77 (74.8)
Age in years				
Range	14-55	14-51	14-35	14-55
Mean ± SD	23.7 ± 9.2	25.6 ± 9.2	23.5 ± 8.1	22.3 ± 9.1
Residence				
Village	27 (13.8)	10 (13.0)	1 (6.3)	16 (15.5)
City	169 (86.2)	67 (87.0)	15 (93.7)	87 (84.5)
Marital status				
Married	34 (17.3)	16 (20.8)	1 (6.3)	17 (16.5)
Unmarried	162 (82.7)	61 (79.2)	15 (93.7)	86 (83.5)
Job status				
Working	43 (21.9)	22 (28.6)	2 (12.5)	19 (18.4)
Not working	153 (78.1)	55 (71.4)	14 (87.5)	84 (81.6)
Highest qualification				
Illiterate/primary school	30 (15.3)	7 (9.1)	2 (12.5)	21 (20.4)
Intermediate school	24 (12.2)	8 (10.4)	2 (12.5)	14 (13.6)
Secondary school	59 (30.1)	25 (32.5)	5 (31.3)	29 (28.2)
University	76 (38.8)	32 (41.5)	7 (43.7)	37 (35.9)
Postgraduate	7 (3.6)	5 (6.5)	0 (0.0)	2 (1.9)
Smoking status				
Never smoke	186 (94.8)	74 (96.1)	15 (93.7)	97 (94.2)
Current smoker	5 (2.6)	1 (1.3)	1 (6.3)	2 (1.9)
Ex-smoker	5 (2.6)	2.6	0 (0.0)	4 (3.9)
Body mass index				
Underweight (<18.49)	38 (19.4)	11 (14.3)	0 (0.0)	27 (26.2)
Normal (18.5-24.99)	86 (43.9)	37 (48.1)	9 (56.2)	40 (38.8)
Overweight (25-29.99)	42 (21.4)	17 (22.1)	4 (25.0)	21 (20.4)
Obese (>30)	30 (15.3)	12 (15.6)	3 (18.8)	15 (14.6)

Nearly half of the patients (90, 46.0%) had been living with diabetes for more than 10 years. Additionally, 25 patients (12.7%) reported a history of diabetic complications, including neuropathy in 12 individuals (6.1%) and retinopathy in 10 (5.1%). A substantial number, 117 patients (59.7%), had experienced hospital admissions due to diabetic ketoacidosis, excluding cases related to diagnosis, with 33 (16.8%) admitted more than three times. Despite the severity of their condition, adherence to the diabetic dietary regimen was suboptimal; 68 patients (34.7%) reported never following the diet, while 97 (49.5%) admitted to sometimes adhering.

Regarding treatment modalities, more than half of the participants (103, 52.5%) were managed with MDIs. In contrast, 49 patients (25.0%) used insulin pumps equipped with sensors (G640 or newer models), and 16 (8.2%) used insulin pumps without sensors. Furthermore, one-quarter of the cohort (49, 25.0%) reported having other chronic diseases, with hypothyroidism being the most common, affecting 24 patients (12.2%). When it came to diabetes self-management, nearly two-thirds (170, 86.9%) stated that they knew how to calculate carbohydrate intake in grams. In addition, 57 patients (29.1%) had attended more than three educator visits in the previous year (Table 2).

**Table 2:** Diabetes-related characteristics of the participants (n = 196). Descriptive statistics are presented as frequencies and percentages. * Denotes not mutually exclusive, indicating that participants could belong to more than one category within that variable.

-	Total (N = 196), N (%)	Insulin pump with sensor (N = 77), N (%)	Insulin pump without sensor (N = 16), N (%)		Multiple dose insulin inject (N = 103), N (%)
Duration of diabetes in years					
≤1	23 (11.7)	1 (1.3)	0 (0.0)		22 (21.4)
>1-5	43 (21.9)	19 (24.7)	2 (12.5)		22 (21.4)
>5-10	40 (20.4)	11 (14.3)	6 (37.5)		23 (22.2)
>10	90 (46.0)	46 (59.7)	8 (50.0)		36 (35.0)
History of diabetic complications					
No	173 (88.3)	74 (96.1)	14 (87.5)		85 (82.5)
Yes	23 (12.7)	3 (3.9)	2 (12.5)		18 (17.5)
Neuropathy	12 (6.1)	2 (2.6)	1 (6.3)		9 (8.7)
Diabetic foot	1 (0.5)	0 (0.0)	0 (0.0)		1 (1.0)
Cardiovascular diseases	3 (1.5)	0 (0.0)	1 (6.3)		2 (1.9)
Nephropathy	3 (1.5)	1 (1.3)	1 (6.3)		1 (1.0)
Retinopathy	10 (5.1)	1 (1.3)	1 (6.3)		8 (7.8)
Non-alcoholic fatty liver	1 (0.5)	0 (0.0)	0 (0.0)		1 (1.0)
History of admission due to diabetic ketoacidosis					
No	79 (40.3)	37 (48.1)	6 (37.5)		36 (35.0)
Yes	117 (59.7)	40 (51.9)	10 (62.5)		67 (65.0)
Once	46 (23.6)	17 (22.1)	0 (0.0)		29 (28.2)
Twice	24 (12.2)	10 (13.0)	0 (0.0)		14 (13.6)
3 times	14 (7.1)	4 (5.2)	1 (6.3)		9 (8.7)
>3 times	33 (16.8)	9 (11.7)	9 (56.3)		15 (14.6)
Compliance with diabetic regimen					
Never	68 (34.7)	33 (42.8)	6 (37.5)		29 (28.2)
Sometimes	97 (49.5)	34 (44.2)	7 (43.7)		56 (54.3)
Always	31 (15.8)	10 (13.0)	3 (18.8)		18 (17.5)
Medications taken*					
Insulin pump with sensor G640/newer	49 (25.0)	_	_	_	
Insulin pump without sensor	16 (8.2)	_	_	_	
Pump with Freestyle Libre	28 (14.3)	_	_	_	
Multiple-dose insulin injection	103 (52.5)	_	_	_	
Metformin	6 (3.1)	_	_	_	
Statins	1 (0.5)	_	_	_	
Anti-hypertensive medications	2 (1.0)	_	_	_	
History of other chronic diseases					
No	147 (75.0)	57 (74.0)	13 (81.2)		77 (74.8)
Yes*	49 (25.0)	20 (26.0)	3 (18.8)		26 (25.2)
Hypertension	5 (2.6)	3 (3.9)	0 (0.0)		2 (1.9)
Cardiac diseases	1 (0.5)	0 (0.0)	0 (0.0)		1 (1.0)
Renal diseases	1 (0.5)	0 (0.0)	0 (0.0)		1 (1.0)
Hypothyroidism	24 (12.2)	12 (15.6)	1 (6.3)		11 (10.7)
Celiac disease	11 (5.6)	2 (2.6)	1 (6.3)		8 (7.8)
Addison's disease	1 (0.5)	0 (0.0)	1 (6.3)		0 (0.0)
Obesity	10 (5.1)	5 (6.5)	1 (6.3)		4 (3.9)
Knowing how to calculate the needed carbohydrates in grams					
No	21 (10.7)	2 (2.6)	1 (6.3)		18 (17.5)
Yes	135 (68.9)	64 (83.1)	13 (81.2)		58 (56.3)
Maybe	40 (20.4)	11 (14.3)	2 (12.5)		27 (26.2)
Number of educators' visits in the last year					
None	50 (25.5)	14 (18.2)	4 (25.0)		32 (31.1)
One	36 (18.4)	14 (18.2)	3 (18.8)		19 (18.4)
Two	31 (15.8)	13 (16.9)	2 (12.5)		16 (15.5)
Three	22 (11.2)	12 (15.6)	1 (6.3)		9 (8.7)
>Three	57 (29.1)	24 (31.2)	6 (37.5)		27 (26.2)

Table 3 illustrates a significant difference in the most recent HbA1c levels among patients with T1DM receiving different insulin therapies. Patients treated with insulin pumps without sensors exhibited the lowest mean HbA1c level at 7.09 ± 0.97, whereas those on MDIs had the highest mean level of 8.35 ± 2.24. The overall p-value was 0.002, with a statistically significant difference observed specifically between these two groups (p = 0.003).

**Table 3:** Comparison of the level of last glycated hemoglobin between different lines of insulin treatment among patients with type 1 diabetes. * One-way analysis of variance. ˚ Indicates a statistically significant difference (p = 0.003). † Indicates a trend toward significance (p = 0.057), based on Tukey's post-hoc test. HbA1c: glycosylated hemoglobin.

	Insulin modality			P-value*
	Insulin pump with sensor (N = 77), Mean ± SD	Insulin pump without sensor (N = 16), Mean ± SD	Multiple dose insulin injection (N = 103), Mean ± SD	
HbA1c %	7.35 ± 1.87˚	7.09 ± 0.97^†^	8.35 ± 2.24˚^†^	0.002

When examining patient satisfaction with their current treatment, several aspects showed notable variation across insulin modalities. These aspects included recent convenience, treatment flexibility, understanding of diabetes, willingness to recommend their treatment to others with T1DM, and continuity of treatment. In all these areas, patients using SAIPs reported the highest satisfaction scores, while those on MDIs reported the lowest. The p-values for these comparisons ranged from 0.004 to less than 0.001, indicating strong statistical significance.

Overall, the highest total satisfaction score was observed among patients using sensor-augmented pumps (Medtronic G640 or newer), averaging 30.19 ± 6.56. In contrast, patients treated with MDIs had the lowest satisfaction score, averaging 23.29 ± 8.05 (p < 0.001) (Table 4).

**Table 4:** Comparison of patient satisfaction between different lines of insulin treatment among patients with type 1 diabetes. One-way analysis of variance. ˚ p < 0.001; p = 0.031; ⱶ p* = 0.003; ⱡ p = 0.015; • p = 0.045 — all based on Tukey’s post-hoc test. Mean satisfaction scores according to treatment modality using the Diabetes Treatment Satisfaction Questionnaire (DTSQs). Source: Diabetes Treatment Satisfaction Questionnaire (DTSQs) © Prof Clare Bradley 9/93. Arabic for Saudi Arabia 28.07.08. Used under license CB1015. Health Psychology Research Unit, Royal Holloway, University of London, Egham, Surrey, TW20 0EX, UK.

	Insulin modality			P-value*
	Insulin pump with sensor (N = 77), Mean (SD)	Insulin pump without sensor (N = 16), Mean (SD)	Multiple dose insulin inject (N = 103), Mean (SD)	
How satisfied are you with your current treatment?	4.96 (1.36)˚	4.63 (1.71)	3.66 (1.89)˚	<0.001
How convenient have you been finding your recent treatment?	4.86 (1.61)^ⱶ^	4.63 (1.67)	4.06 (1.60)^ⱶ^	0.004
How flexible have you been finding your recent treatment?	5.04 (1.28)˚	4.31 (1.89)	3.81 (1.49)˚	<0.001
How satisfied are you with your understanding of your diabetes?	5.14 (1.23)^ⱶ^	5.13 (1.71)	4.38 (1.70)^ⱶ^	0.003
Would you recommend this form of treatment to someone else having similar diabetes?	5.47 (1.13)˚	5.25 (1.34)^ⱡ^	4.09 (1.82)˚^ ⱡ^	<0.001
How satisfied would you be to continue with your present form of treatment?	4.73 (1.63)˚	4.50 (1.97)^•^	3.30 (1.99)˚^•^	<0.001
Overall satisfaction	30.19 (6.56)˚	28.44 (8.18)*	23.29 (8.05)˚*	<0.001

The study revealed that patients receiving MDIs reported the highest perceived frequency of hyperglycemia. In contrast, those treated with SAIPs experienced the lowest frequency of hyperglycemia. This difference was statistically significant (p = 0.018). However, when examining the perceived frequency of hypoglycemia, no significant differences were found among the various insulin modalities (Table 5).

**Table 5:** Comparison of hyper-/hypoglycemic experience between different lines of insulin treatment among patients with type 1 diabetes. * Statistically significant difference between specific insulin treatment groups (p = 0.018) according to Tukey’s post-hoc test. ⱶ One-way analysis of variance. P = 0.018 (Tukey’s post-hoc test).

	Insulin modality			P-value^ⱶ^
	Insulin pump with sensor (N = 77), Mean (SD)	Insulin pump without sensor (N = 16), Mean (SD)	Multiple dose insulin injection (N = 103), Mean (SD)	
How often have you felt that your blood sugars are unacceptably high recently?	3.06 (1.71)*	3.38 (1.54)	3.75 (1.62)*	0.018
How often have you felt your blood sugars have been unacceptably low recently?	2.70 (1.88)	3.13 (1.86)	3.25 (1.75)	0.129

Additionally, a significant negative correlation was observed between patient age and the most recent HbA1c level (r = -0.155, p = 0.030), indicating that younger patients tended to have higher HbA1c values. Moreover, patients who had developed diabetic complications exhibited significantly higher HbA1c levels compared to those without complications (8.84 ± 2.20 vs. 7.72 ± 2.03, p = 0.015). Importantly, patients who were knowledgeable about calculating dietary carbohydrates in grams demonstrated better glycemic control, with lower HbA1c levels than those lacking this knowledge (7.53 ± 1.98 vs. 8.48 ± 2.22, p = 0.005; Table 6).

**Table 6:** Factors associated with glycemic control among patients with type 1 diabetes mellitus. HbA1c: glycosylated hemoglobin. * Student’s t-test. ** One-way analysis of variance (ANOVA). † Pearson correlation test.

Variables	Last HbA1c, Mean (SD)	P-value
Gender		
Male	7.77 (1.82)	0.769*
Female	7.88 (2.16)	
Age in years	r = -0.155	0.030^†^
Residence		
Village	7.70 (1.74)	0.689*
City	7.88 (2.13)	
Marital status		
Married	7.84 (2.37)	0.956*
Unmarried	7.86 (2.02)	
Job status		
Working	7.57 (1.86)	0.321*
Not working	7.93 (2.14)	
Highest qualification		
Illiterate/primary school	8.41 (1.66)	
Intermediate school	8.38 (2.18)	
Secondary school	8.0 (2.28)	
University	7.42 (2.04)	
Postgraduate	7.11 (0.91)	0.087**
Smoking status		
Never smoke	7.85 (2.07)	
Current smoker	7.16 (1.21)	
Ex-smoker	9.78 (2.59)	0.086**
Duration of diabetes in years		
≤1	8.90 (2.97)	
>1-5	7.6 (1.86)	
>5-10	7.85 (1.65)	
>10	7.70 (2.03)	0.073**
History of diabetic complications		
No	7.72 (2.03)	
Yes	8.84 (2.20)	0.015*
History of admission due to diabetic ketoacidosis		
No	7.52 (1.85)	
Yes	8.08 (2.20)	0.069*
Compliance with the diabetic regimen		
Never	8.22 (2.29)	
Sometimes	7.51 (1.78)	
Always	7.49 (1.81)	0.052**
History of other chronic diseases		
No	7.86 (2.22)	
Yes	7.82 (1.60)	0.893*
Knowing how to calculate the needed carbohydrates in grams		
No	8.48 (2.22)	
Yes	7.53 (1.98)	
May be	8.61 (2.10)	0.005*
Number of educators' visits in the last year		
None	7.60 (1.78)	
One	7.88 (2.66)	
Two	7.53 (1.53)	
Three	7.50 (1.72)	
>Three	8.38 (2.25)	0.220**

As shown in Table 7, patients using SAIPs exhibited significantly lower HbA1c levels compared to those relying on MDIs, even after adjusting for confounding variables. Age appeared to have a modest influence on HbA1c, explaining approximately 7% of its variability (r² = 0.073). In contrast, adherence to the dietary regimen and knowledge of carbohydrate counting in grams were not significantly associated with HbA1c levels in this analysis.

**Table 7:** Best-fitting multiple linear regression model for glycosylated hemoglobin level among patients with type 1 diabetes mellitus. R² = 0.073; adjusted R² = 0.063. ANOVA: F = 7.593, p = 0.001. Variables entered and excluded: adherence to diet regimen and knowledge of how to calculate required carbohydrate intake in grams.

	Unstandardized coefficients		Standardized coefficients	t-test	p-value	95% CI for B	
	B	Std. error				Lower	Upper
Constant	7.487	0.548		13.659	<0.001		
Age in years	-0.03	0.016	-0.132	-1.887	0.061	-0.061	0.001
Insulin pump with sensors (reference: multiple insulin injections)	0.365	0.114	0.223	3.195	0.002	0.14	0.59

The study also found that patients who were proficient in calculating carbohydrates reported higher treatment satisfaction than those who were not. Specifically, the satisfaction score among patients knowledgeable in carbohydrate counting was 27.24 ± 7.81, compared to 21.71 ± 9.93 for those without this skill (p = 0.015). Furthermore, the frequency of visits to diabetes educators had a significant effect on satisfaction levels. Patients who attended more than three educator visits achieved the highest satisfaction score of 29.12 ± 7.14, whereas those with no visits reported the lowest score of 23.72 ± 9.16 (p = 0.014; Table 8).

**Table 8:** Factors associated with satisfaction among patients with type 1 diabetes mellitus. * Student’s t-test. ** One-way analysis of variance (ANOVA). † Pearson correlation test. Mean satisfaction scores according to treatment modality using the Diabetes Treatment Satisfaction Questionnaire (DTSQs). Data are presented as mean ± SD. P-values were calculated using one-way ANOVA. Source: Diabetes Treatment Satisfaction Questionnaire (DTSQs) © Prof Clare Bradley 9/93. Arabic for Saudi Arabia 28.07.08. Used under license CB1015. Health Psychology Research Unit, Royal Holloway, University of London, Egham, Surrey, TW20 0EX, UK.

Variables	Patient`s satisfaction score, Mean (SD)	P-value
Gender		
Male	26.52 (8.43)	
Female	26.39 (8.13)	0.926*
Age in years	r = 0.078	0.276^†^
Residence		
Village	26.26 (7.72)	
City	26.45 (8.28)	0.911*
Marital status		
Married	27.47 (7.34)	
Unmarried	26.20 (8.35)	0.413*
Job status		
Working	27.02 (7.92)	
Not working	26.25 (8.27)	0.588*
Highest qualification		
Illiterate/primary school	24.23 (9.73)	
Intermediate school	26.17 (6.59)	
Secondary school	26.12 (8.51)	
University	27.22 (7.96)	
Postgraduate	30.57 (2.70)	312**
Smoking status		
Never smoke	26.56 (8.21)	
Current smoker	25.20 (9.47)	
Ex-smoker	22.60 (5.68)	0.536**
Duration of diabetes in years		
≤1	22.48 (8.73)	
>1-5	27.00 (7.89)	
>5-10	26.45 (7.93)	
>10	27.14 (8.14)	0.099**
History of diabetic complications		
No	26.79 (8.05)	
Yes	23.65 (8.86)	0.084*
History of admission due to diabetic ketoacidosis		
No	27.30 (7.94)	
Yes	25.83 (8.32)	0.217*
Compliance with diabetic regimen		
Never	26.87 (9.04)	
Sometimes	25.67 (7.76)	
Always	27.81 (7.46)	0.387**
History of other chronic diseases		
No	26.46 (8.13)	
Yes	26.33 (8.42)	0.0924*
Knowing how to calculate the needed carbohydrates in grams		
No	21.71 (9.93)	
Yes	27.24 (7.81)	
May be	26.13 (7.78)	0.015**
Number of educators' visits in the last year		
None	23.72 (9.16)	
One	25.44 (8.37)	
Two	26.55 (7.78)	
Three	27.00 (6.93)	
>Three	29.12 (7.14)	0.014**

As presented in Table 9, patients using SAIPs reported significantly higher satisfaction levels compared to those relying on MDIs, even after adjusting for confounding variables. In addition, an increased number of visits to diabetes educators was positively associated with greater patient satisfaction. Together, these factors explained approximately 20% of the variability in satisfaction scores (r² = 0.202).

**Table 9:** Best-fitting multiple linear regression model for patient satisfaction among patients with type 1 diabetes mellitus. R² = 0.202; adjusted R² = 0.194. ANOVA: F = 24.446, p < 0.001. Variable entered: knowledge of how to calculate the required carbohydrate intake in grams. Mean satisfaction scores according to treatment modality using the Diabetes Treatment Satisfaction Questionnaire (DTSQs). Data are presented as mean ± SD. P-values were calculated using one-way ANOVA. Source: Diabetes Treatment Satisfaction Questionnaire (DTSQs) © Prof Clare Bradley 9/93. Arabic for Saudi Arabia 28.07.08. Used under license CB1015. Health Psychology Research Unit, Royal Holloway, University of London, Egham, Surrey, TW20 0EX, UK.

	Unstandardized coefficients		Standardized coefficients	t-test	P-value	95% CI for B	
	B	Std. error				Lower	Upper
Constant	31.286	1.533		20.412	<0.001		
Insulin pump with sensors (reference: multiple insulin injections)	-3.25	0.558	-0.378	-5.827	<0.001	-4.35	-2.15
Number of educators' visits in the last year (reference: No)	1.034	0.336	0.199	3.076	0.002	0.37	1.7

## Discussion

Managing T1DM presents ongoing challenges for both healthcare providers and patients. The unpredictable fluctuations in blood glucose levels, coupled with a high rate of poor glycemic control, complicate effective disease management. To address these issues, this study primarily aimed to compare glycemic control and patient satisfaction across different insulin delivery methods, specifically, insulin pumps with or without sensors (operating in suspended low and pre-low modes) and MDIs among individuals with T1DM.

After conducting univariate and multivariate analyses on patient data, the study found that HbA1c levels, a key indicator of glycemic control, were significantly better in patients using SAIPs compared to those receiving MDI therapy. This improvement likely stems from the enhanced precision of insulin delivery provided by pump therapy, which allows for better dose adjustments in response to varying activity levels [12,13]. Supporting this, prior research from Saudi Arabia demonstrated HbA1c improvements, particularly among female patients and those with a shorter duration of T1DM after six months of treatment [14]. However, unlike these studies, our analysis did not find any significant correlation between glycemic control and either patient gender or duration of diabetes.

Several international studies corroborate these findings. For example, Hermanides et al. randomized patients with T1DM and HbA1c levels ≥ 8.2% to continue MDI therapy or switch to sensor-augmented pump therapy, reporting a significant reduction in HbA1c in the pump group [15]. Similarly, Bergenstal et al. observed that sensor-augmented pump therapy reduced HbA1c to 7.5% from baseline, compared to 8.1% in the MDI group, with a greater proportion achieving target glycemic control (HbA1c < 7%) in the pump group [8]. Furthermore, Buse et al. found that while sensor glucose values were comparable between the two groups at HbA1c ≥ 6.5%, patients on sensor-augmented pumps had lower glucose variability when HbA1c was below 8% [7].

Beyond glycemic control, our study also demonstrated improved patient satisfaction with insulin pumps equipped with sensors, even after controlling for confounders in multivariate analysis. This aligns with both local [14] and international research [12,13,16]. Several factors may contribute to this increased satisfaction, including reduced physical and dietary restrictions [17], as well as enhanced patient confidence and self-efficacy in managing diabetes with sensor technology [18], particularly among children [19].

Consistent with these findings, Hussain et al. reported higher satisfaction scores among sensor-augmented pump users compared to those on MDI, citing greater ease of use, overall treatment satisfaction, and confidence in disease management [16]. The flexibility provided by sensor-augmented pumps allows patients to make real-time adjustments in response to exercise, meal timing and composition, and variable basal insulin needs [17,20].

Another important factor influencing patient satisfaction was the frequency of visits to diabetes educators. Our multivariate analysis confirmed that patients who had more than three educator visits reported the highest satisfaction scores, underscoring the critical role of diabetes education in treatment outcomes. Interestingly, while some studies from Western countries have noted lower satisfaction among female insulin pump users due to device visibility [21,22], our study found no gender differences. This discrepancy may be explained by cultural practices in Saudi Arabia, where many women wear the abaya, a full-length outer garment that conceals the device [14].

In terms of glycemic events, patients on MDI reported the highest perceived frequency of hyperglycemia, whereas those using sensor-augmented pumps reported the lowest. No significant differences were observed between treatment modalities regarding perceived hypoglycemic symptoms. Supporting this, a Saudi study found that insulin pumps with sensors significantly reduced both hyperglycemia and hypoglycemia in female patients after six months, particularly in those with a shorter duration of T1DM [14]. Similarly, Bergenstal et al. reported no significant difference in severe hypoglycemia rates between patients treated with sensor-augmented pumps and those on MDI [8].

This study does have several limitations. First, it was conducted at a single center, although this center serves as a major referral hub for T1DM patients across Riyadh. Second, the sample size was insufficient for some treatment groups, particularly patients using insulin pumps without sensors. Third, as this was an observational study, the inability to establish cause-and-effect relationships must be acknowledged. Despite these limitations, our findings have important clinical implications for T1DM management and highlight the need for further research on newer hybrid closed-loop pump technologies within Saudi Arabia.

## Conclusions

Treating patients with T1DM using insulin pumps equipped with sensors has demonstrated clear advantages over MDIs, particularly in terms of glycemic control and treatment satisfaction. Patients using SAIPs not only achieved better regulation of blood glucose levels but also reported greater overall satisfaction with their treatment regimen. Furthermore, the perceived frequency of hyperglycemic episodes was notably lower among this group, suggesting improved real-time glucose management.

In addition to the treatment modality, the frequency of follow-up with diabetes educators played a significant role in patient outcomes. Individuals who attended more frequent educational sessions expressed higher satisfaction with their medications, highlighting the importance of continued patient support and education in chronic disease management. These findings collectively underscore the value of advanced insulin delivery systems and the supportive role of healthcare professionals in optimizing care for individuals with T1DM.

## Figures and Tables

**Figure 1:**
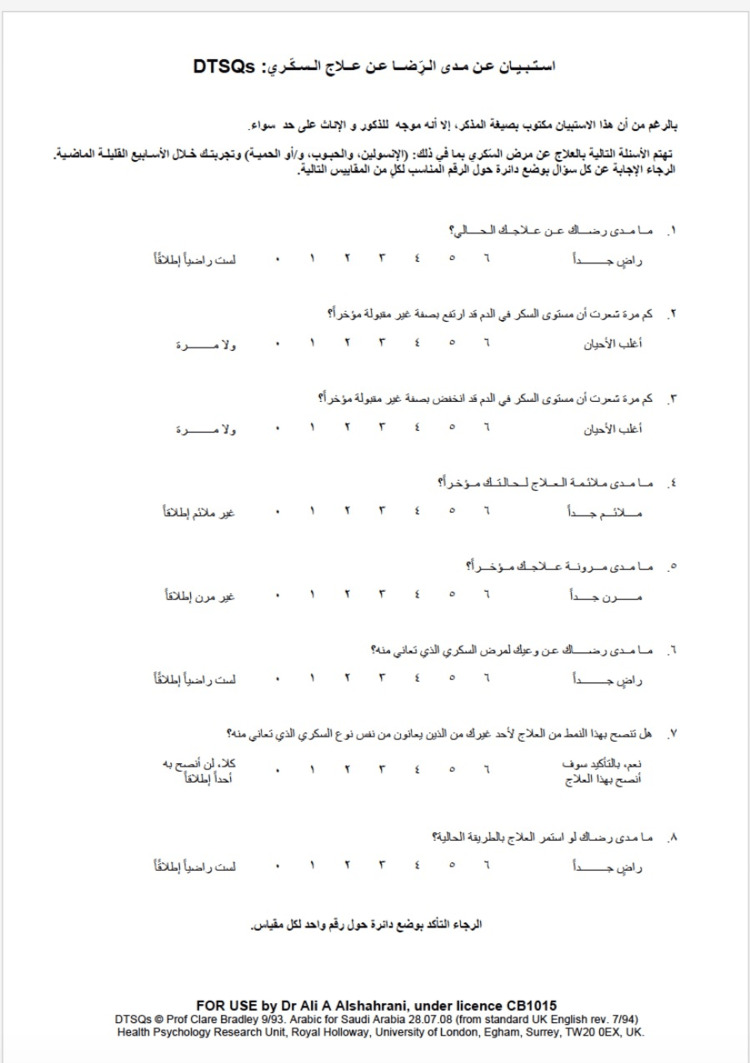
Arabic version of the Diabetes Treatment Satisfaction Questionnaire (DTSQs). Use of the questionnaire was authorized under license (CB1015) granted to Dr. Ali A. Alshahrani by the Health Psychology Research Unit, Royal Holloway, University of London.

**Figure 2:**
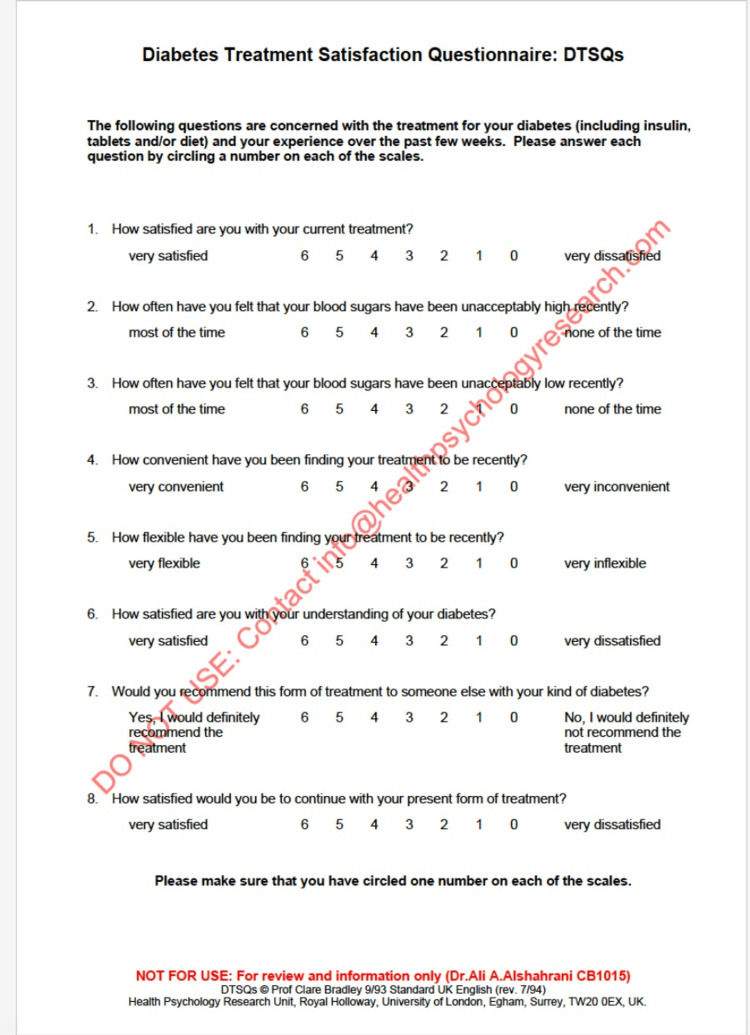
English version of the Arabic version of the Diabetes Treatment Satisfaction Questionnaire (DTSQs). Use of the questionnaire was authorized under license (CB1015) granted to Dr. Ali A. Alshahrani by the Health Psychology Research Unit, Royal Holloway, University of London.
